# Vanadium Stress-Driven Microbial Acclimation Enhances Biological Denitrification in Recycling of Vanadium-Containing Industrial Wastewater

**DOI:** 10.3390/microorganisms13051003

**Published:** 2025-04-27

**Authors:** Yihuan She, Yimin Zhang, Qiushi Zheng, Zhenlei Cai, Yue Wang, Nannan Xue

**Affiliations:** 1 School of Resources and Environmental Engineering, Wuhan University of Science and Technology, Wuhan 430081, China; jiheweiyi712812@163.com (Y.S.);; 2 State Environmental Protection Key Laboratory of Mineral Metallurgical Resources Utilization and Pollution Control, Wuhan 430081, China; 3 Collaborative Innovation Center of Strategic Vanadium Resources Utilization, Wuhan 430081, China; 4 Hubei Provincial Engineering Technology Research Center of High Efficient Cleaning Utilization for Shale Vanadium Resource, Wuhan 430081, China

**Keywords:** vanadium, ammonia–nitrogen wastewater, denitrification, *Pseudomonas*, material transport, AMO specific enzyme

## Abstract

Recirculation in vanadium mining enhances resource efficiency but risks ammonia nitrogen (NH_3_-N) accumulation, severely compromising leaching yields. To address this bottleneck, we developed a bioaugmentation strategy using *Pseudomonas* sp. S.P-1 acclimated to vanadium stress. Under optimized conditions (sodium citrate as a carbon source, C/N = 5, 5% inoculum, and pH = 8), the strain achieved exceptional NH_3_-N (2000 mg·L^−1^) removal (>99.25% within 16 days; residual NH_4_^+^ < 15 mg·L^−1^), 12.7% higher than the original bacteria. Mechanistic studies revealed that vanadium exposure triggered dual adaptive responses: enhanced biosorption via the stimulated synthesis of extracellular polymeric substances (EPS) enriched with negatively charged functional groups (C=O, -COOH-, and C-N), improving NH_4_^+^ adsorption capacity, and metabolic activation via an elevated transmembrane electrochemical potential and an accelerated substrate uptake due to cell membrane permeability, while up-regulation of ammonia monooxygenase (AMO) activity (123.11%) facilitated efficient NH_4_^+^→NH_2_OH conversions. Crucially, this bio-process enabled simultaneous NH_3_-N degradation (89.2% efficiency) and vanadium recovery, demonstrating its dual role in pollution control and critical metal recycling. By integrating microbial resilience with circular economy principles, our strategy offers a scalable prototype for sustainable vanadium extraction, aligning with low-carbon metallurgy demands in clean energy transitions. This study investigated the ability of vanadium stress to enhance microbial ammonia nitrogen metabolism, and by acclimatizing S.P-1 to vanadium-containing solutions, we aimed to address the dual problems of NH_3_-N accumulation and vanadium toxicity in wastewater recirculation.

## 1. Introduction

Ore mining and metal extraction not only serve as the cornerstone of modern industry but also act as pivotal drivers for technological revolution, energy transition, and sustainable development [1]. Hydrometallurgy and pyrometallurgy currently represent the two primary methodologies for metal extraction. Due to the complexity of mineral compositions, diverse smelting processes are employed, resulting in wastewater streams with heterogeneous characteristics. Notably, the introduction of ammonium salts during these processes generates wastewater containing excessive ammonia–nitrogen concentrations that far exceed regulatory discharge limits. Even when recycled, such ammonia-laden effluents impair subsequent extraction efficiency [2]. Consequently, targeted ammonia–nitrogen removal is imperative to provide theoretical underpinnings for enhancing metallurgical productivity and achieving sustainable practices.

Taking the vanadium industry as an illustrative example, vanadium—a critical strategic rare metal—plays an irreplaceable role in modern chemical engineering, metallurgy, renewable energy, and aerospace applications, with its global significance widely acknowledged [3]. Vanadium extraction processes generate substantial desilication wastewater (termed desilication mother liquor), which typically contains residual vanadium precipitates or dissolved vanadium species that fail to completely crystallize during precipitation reactions [4]. These residual vanadium components retain high recovery value, prompting industrial practices to recycle the mother liquor back to leaching or precipitation stages for vanadium and reagent recovery. However, the elevated ammonia–nitrogen content in the recycled mother liquor severely compromises subsequent vanadium extraction efficiency and destabilizes operational processes [5].

The introduction of ammonium salts in vanadium extraction processes results in wastewater containing elevated ammonia–nitrogen (NH_4_^+^-N) levels following vanadium precipitation. The ammonia-laden wastewater generated from industrial vanadium production has accumulated significantly. The NH_4_^+^-N concentrations in such wastewater vary depending on precipitation methods, typically ranging from 10,000 to 30,000 mg·L^−1^. Upon recycling this wastewater back into the extraction process, the enriched ammonium ions (NH_4_^+^-N) tend to form complexation reactions with fluoride ions (F^−^), thereby hindering F^−^’ s role in facilitating the lattice decomposition of vanadium-bearing mica in shale. Furthermore, NH_4_^+^ readily reacts with sulfate ions (SO_4_^2−^) and aluminum ions (Al^3+^) to precipitate as ammonium aluminum sulfate (NH_4_Al(SO_4_)_2_·12H_2_O), causing pipeline scaling and blockage. These interactions markedly inhibit vanadium extraction efficiency during the recycled leaching process and adversely affect subsequent precipitation performance.

At the present, the treatment methods for high concentration of ammonia–nitrogen wastewater in industry include three major categories: physicochemical, advanced oxidation and biological [6,7]. Physicochemical methods are prone to secondary pollution (waste gas, residue), sensitive to pH, high cost, and high consumption of chemicals. Advanced oxidation method is prone to produce large amounts of toxic substances, high energy consumption and high cost. Traditional biochemical method has a long process, a large reactor and a large footprint. The discovery of heterotrophic nitrification–aerobic denitrification (HNAD) in new biological nitrogen removal techniques avoids these problems. The HNAD process has several advantages over conventional biological nitrogen removal processes: (1) the process achieves nitrogen removal and COD removal under fully aerobic conditions [8]; (2) the alkalinity consumed during the nitrification process is compensated for through the denitrification process at the same time [9]; (3) it saves space and cost [8]; and (4) it reduces energy consumption and carbon emissions [10].

Ammonia–nitrogen wastewater in the vanadium extraction from vanadium-bearing shale industry contain high concentrations of vanadium. Vanadium is an essential trace element for some microorganisms. It has been shown that a decrease in some metal-tolerant bacteria (e.g., *Fusobacterium actinomycetemcomitans*) was observed in bioreactors with high vanadium (V) concentrations, whereas *Pseudomonas aeruginosa* has a high abundance in culture. However, vanadium can be toxic to microorganisms and alter microbial activity when the vanadium content exceeds a certain concentration [11]. In groundwater vanadium remediability studies, the higher the vanadium concentration in the aquatic environment, the lower the abundance and diversity of the microbial community [12]. In a study on the removal of vanadium from a microbial fuel cell system, vanadium promotes the formation of microbial membranes on the electrodes and increases the ability of microorganisms to convert glucose at a certain concentration, but high concentrations of vanadium will inhibit the growth of microorganisms [13]. Currently, vanadium research on microorganisms focuses on effects on community abundance and diversity, and lacks exploration of the effects of vanadium on specific microbial functions.

*Pseudomonas* sp. also has a number of applications in the HNAD process. It has been found that *Pseudomonas* WL20-3 removed 100% of the initial ammonia–nitrogen concentration of 240 mg L^−1^ at 4 °C [14]. *Pseudomonas* Y1 was able to remove ammonia–nitrogen, phosphate, and calcium synchronously with removal rates of 92.04%, 99.98%, and 83.4%, respectively [15]. *Pseudomonas* F2 showed good growth performance but lower ammonia removal of 41.23% when the ammonia concentration was 500 mg L^−1^, which increased to 94.92% after optimizing the carbon source and dissolved oxygen level [16]. Most of the studies have focused on low to medium concentration ammonia–nitrogen wastewater, which has the advantages of rapid growth, adaptability, and high ammonia–nitrogen removal efficiency. However, there is a lack of research on the purification of high concentration ammonia–nitrogen wastewater exceeding 500 mg L^−1^.

In response to the dual challenges of high ammonia–nitrogen (NH_4_^+^-N) accumulation and inhibition of microbial activity by vanadium toxicity in vanadium industrial wastewater recycling, the present study constructed a bioaugmentation strategy by domesticating S.P-1 through vanadium stress, aiming to break through the limitations of the traditional biological method for the treatment of vanadium-containing, high ammonia–nitrogen wastewater (2000 mg·L^−1^). Although ammonia removal is the core target, the presence of vanadium significantly affects microbial metabolic pathways, and previous studies have mostly explored nitrogen removal efficiency or metal tolerance in isolation, neglecting the bidirectional regulatory mechanisms of vanadium in biological ammonia metabolism. Therefore, this study was conducted to reveal the mechanism of the vanadium-enhanced denitrification performance of S.P-1, from the perspective of changes in S.P-1 characterization and enzymes, and to fill the theoretical gaps in the mechanisms of vanadium and microbial interactions.

## 2. Materials and Methods

### 2.1. Ammonia–Nitrogen Wastewater

**Figure 1:**
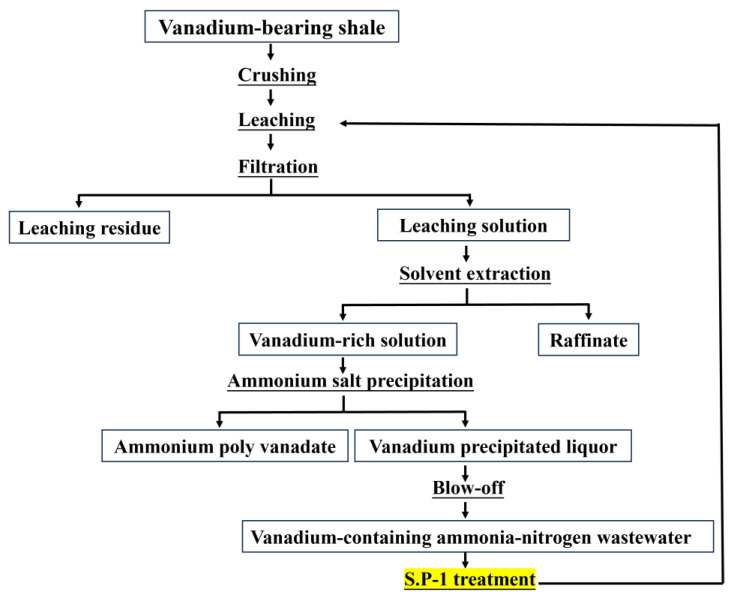
Simplified process diagram for vanadium ammonia–nitrogen wastewater treatment.

The source of vanadium-containing ammonia–nitrogen wastewater is shown in Figure 1. The ammonia–nitrogen wastewater’s pH is 8, and the elemental composition is shown in Table 1.

### 2.2. Main Medium

Luria–Bertani (LB) broth medium, containing (per liter, pH = 7.0) yeast extract, 5.0 g; peptone, 10.0 g; and NaCl, 10.0 g, was used for screening and purification of the strains.

The denitrification medium (denitrification medium-1) (DM-1) for the nitrite reduction studies (per liter) contained NaNO_2_, 0.3 g; Na_2_SO_4_, 4.737g; K_2_SO_4_, 0.1743 g; MgSO_4_·7H_2_O, 0.1873 g; CaSO_4_, 0.34 g; and NH_4_VO_3_, 0.2204 g.

The denitrification medium (denitrification medium-2) (DM-2) for the nitrate reduction studies (per liter) included the following: NaNO_3_, 0.28 g; Na_2_SO_4_, 4.737 g; K_2_SO_4_, 0.1743 g; MgSO_4_·7H_2_O, 0.1873 g; CaSO_4_, 0.34 g; and NH_4_VO_3_, 0.2204 g.

The Simultaneous Nitrification and Denitrification Mixed Medium (SNDM-1&2) (per liter) included the following: (NH_4_)_2_SO_4_, 0.37 g; NaNO_2_, 0.15 g (or (NH_4_)_2_SO_4_, 0.37 g, and NaNO_3_, 0.14 g); Na_2_SO_4_, 4.737 g; K_2_SO_4_, 0.1743 g; MgSO_4_·7H_2_O, 0.1873 g; CaSO_4_, 0.34 g; and NH_4_VO_3_, 0.2204 g.

Solid medium was prepared by adding 1.5% agar to LB. All chemicals were of analytical grade. Each medium was placed in an autoclave at 121 °C for 20 min before use.

### 2.3. Source and Identification of Strains

The *Pseudomonas* sp. (S.P) strains used in this study were purchased from the CGMCC China General Microbial Strain Collection and Management Center (Shanghai, China).

Genomic DNA was extracted using the TIANGEN Bacterial Genomic DNA Extraction Kit (Tiangen Biotech, Beijing China) and amplified using the 16S rRNA gene universal primers 27F (5′-AGAGTTTGATCCTGGCTCAG-3′) and 1492R (5′-TACGGCTACCTTGTTACGACTT-3′). Sequencing was performed by Shanghai Fuda Testing Technology Group Corporation. The sequencing results were compared and analyzed using the Basic Local Alignment Search Tool (BLAST) of the National Center for Biotechnology and Information (https://www.ncbi.nlm.nih.gov/BLAST/Blast.cgi, accessed on 24 April 2025).

### 2.4. Domestication of Bacteria

A total of 5 mL of S.P protobacterium in the logarithmic phase was transferred into 100 mL of wastewater with a vanadium concentration of 100 mg L^−1^ and an ammonia–nitrogen concentration of 100 mg L^−1^, and the bacterial concentration in the medium was measured periodically. After the bacterial growth curves in the culture solution reached a stable period, the bacteria were inoculated into LB solid medium, and the growth of the colonies in the medium was observed. The colony morphology of *Pseudomonas* strains on LB plates was round and milky white, with a smooth and opaque surface. When S.P colonies appeared on the solid medium, the colonies were randomly and evenly selected for inoculation into a simulation of waste liquids. This was repeated when bacterial growth entered the logarithmic phase.

### 2.5. Single Factor Experimental Design Affecting the Performance of Heterotrophic Deamination

A one-way experiment was used to optimize the culture conditions, including carbon source, carbon-to-nitrogen ratio (C/N ratio), pH, and inoculum amount.

All of the above experiments are conducted three times in 250 mL conical flasks containing 100 mL of a sterile simulation of waste liquids. Periodic samples were taken to measure OD_600_ and pH, and the supernatant was used to determine NH_4_^+^-N, NO_2_-N, NO_3_-N, and chemical oxygen demand (COD).

### 2.6. Assessment of Aerobic Denitrification Capability

Heterotrophic nitrification capacity of *Pseudomonas aeruginosa*-domesticated strains was assessed by taking 1 mL of the bacterial suspension of the domesticated bacteria and inoculating it in 100 mL of simulation of waste liquids in a 250 mL conical flask.

The aerobic denitrification performance of *Pseudomonas aeruginosa*-domesticated strains was investigated using DM-1 and DM-2 media spiked with 100 mg L^−1^ NO_3_^−^-N and 100 mg L^−1^ NO_2_^−^-N, respectively. SNDM-1&2 media supplemented with both 100 mg L^−1^ NH_4_^+^-N and 100 mg L^−1^ NO_3_^−^-N (100 mg L^−1^ NH_4_^+^-N and 100 mg L^−1^ NO_2_^−^-N) were used to study the denitrification ability of *Pseudomonas* aeruginosa-domesticated strains under combined nitrogen sources.

The above experiments were incubated under sodium citrate as the sole carbon source, 30 °C, 200 rpm, pH = 8.0, and C/N = 5, and samples were taken periodically from the bottles to determine OD_600_, NH_4_^+^-N, NO_2_^−^-N, NO_3_^−^-N, COD, and pH. All experiments were performed in triplicate.

### 2.7. Enzyme Assay

The activities of nitrate reductase (NR), nitrite reductase (NiR), hydroxylamine oxidase (HAO), and ammonia monooxygenase (AMO) of S.P-1 were tested by Shanghai Fuda Testing Technology Group Corporation (Shanghai, China).

The bacteria were first separated from the medium by centrifugation, and then the cell-free extracts were washed three times with 0.01 M potassium phosphate buffer solution (pH 7.4). Bacteria in suspension were lysed by ultrasound and then centrifuged at 4 °C and 12,000 rpm for 5 min to remove intact cells and cellular debris [17]. The activities of NR and NIR were measured using NO_2_^−^ formed by NO_3_^−^ reduction and the reduction in NO_2_^−^, respectively [17]. AMO was measured by oxidation of NH_4_^+^ [18]. HAO activity was analyzed by the reduction reaction of potassium ferricyanide at 400 nm [19]. Enzyme activity (U) was defined as the concentration of enzymes that catalyzes the conversion of 1 μmol of substrate to product in 1 min [20].

### 2.8. PCR Amplification of Nitrification and Denitrification Genes in Pseudomonas Aeruginosa

Performed by the Shanghai Fuda Testing Technology Group Corporation (Shanghai, China), PCR amplification was used to detect the related nitrogen metabolism genes in S.P and S.P-1, and the amplification primers are shown in Table 2.

### 2.9. Analysis Methods

The pH of the solution is measured using a pH meter (PHS-3C Shanghai Yidian Scientific Instrument Co., Ltd., Shanghai, China) [21]. Nitrate nitrogen is measured using UV spectrophotometry, and nitrite nitrogen is measured spectrophotometrically [21]. Ammonia–nitrogen is detected using Aptar Ion Chromatograph (940 Professional, Shanghai, China) [21]. Bacterial membrane potential is measured using a flow cytometer of Guava EasyCyte type from Luminex, Switzerland [22]. X-ray photoelectron diffraction energy spectroscopy (K-Alpha) from ThermoScientific, Waltham, MA, USA, is used to detect and analyze the functional groups in the EPS of S.P and S.P-1 [22].

## 3. Results and Discussion

### 3.1. Factors Affecting the Ammonia–Nitrogen Removal Performance of S.P-1

#### 3.1.1. Effect of Carbon Sources

**Figure 2:**
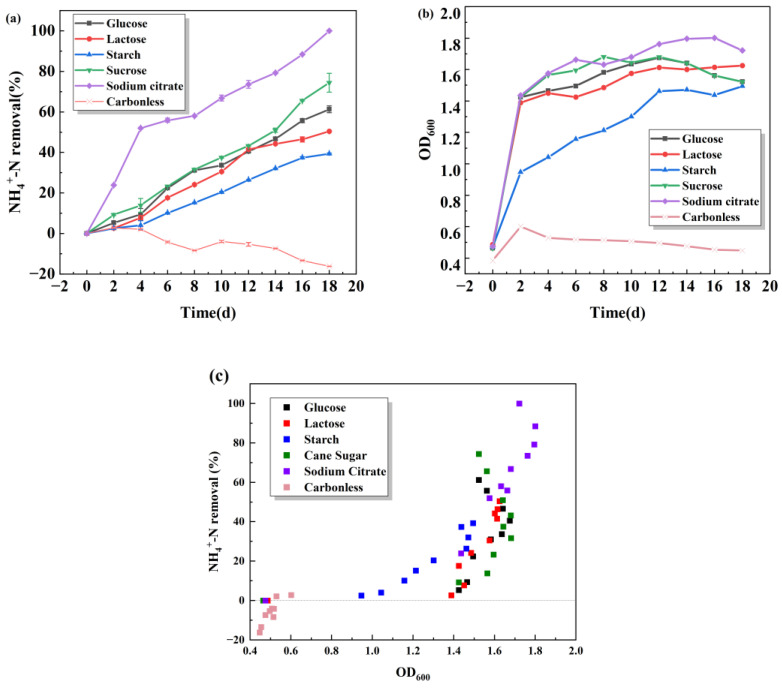
Effects of carbon sources on NH_4_^+^-N removal (**a**) and OD_600_ (**b**) and scatter plot of NH_4_^+^-N removal and OD_600_ (**c**).

In the growth and reproduction process of heterotrophic S.P-1 microorganisms, it is necessary to utilize an external carbon source to provide the required energy and material basis for normal growth and life activities. There are differences in the chemical composition of different carbon sources, and so the utilization degree of carbon sources by microorganisms is different. The most suitable carbon source is conducive to the growth of bacterial strains and the best denitrification effect, thereby shortening denitrification time and reducing medication costs. As seen in Figure 2b, S.P-1 grows better with additional carbon sources than without them, indicating that S.P-1 utilizes external carbon sources for reproduction and heterotrophic nitrification. The growth of S.P-1 in all five carbon sources shows a trend from rapid proliferation to remaining stable. In Figure 2a, different carbon sources lead to varying degrees of ammonia–nitrogen removal as shown by sodium citrate > sucrose > glucose > lactose > starch. Starch, the most unfavorable for S.P-1, removes ammonia–nitrogen, which is probably due to the difficulty of S.P-1 in utilizing macromolecules. Some research has shown that strains are more likely to utilize organic carbon sources and small molecule carbon sources [23]. Sodium citrate is the most favorable for S.P-1 and removes ammonia–nitrogen due to the simple and small molecular structure of organic acids, which are more favorable than sugars and can be utilized directly, and the sodium citrate participates in the tricarboxylic acid cycle directly [24]. After the fourth day, the ammonia–nitrogen removal rate of sodium citrate increased gradually and reached 100% by the 18th day, while the ammonia–nitrogen removal rate of other experimental groups was lower. The above results showed that S.P-1 utilizes sodium citrate as a carbon source well and has a strong environmental adaptability. Considering the cell growth and heterotrophic nitrification ability, sodium citrate is chosen as the only carbon source for the next study.

#### 3.1.2. Effect of C/N Ratio

**Figure 3:**
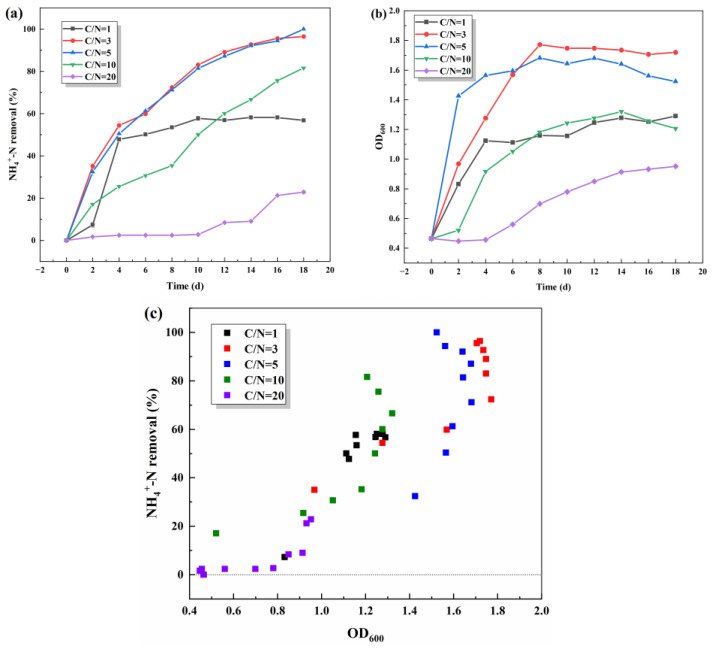
Effects of C/N on NH_4_^+^-N removal (**a**) and OD_600_ (**b**) and scatter plot of NH_4_^+^-N removal and OD_600_ (**c**).

C/N is the mass ratio of the total content of carbon to the total content of nitrogen in the medium, which is an important indicator for the biological treatment of nitrogenous wastewater, and the selection of a suitable C/N is conducive to the microorganisms being able to play a maximum effect. The effect of C/N on denitrification performance was investigated by varying only C/N (1, 3, 5, 10, and 20) in the wastewater. As can be seen from the Figure 3c, the growth of the strains coincided better with the removal of NH_4_^+^-N under different C/N. With the time extension, the growth of the strains all showed a trend of increasing and then stabilizing in Figure 3b. At C/N = 3 and C/N = 5, the number of bacteria grew faster in the pre-culture period, and the rate of ammonia–nitrogen removal remained essentially the same. On the 18th day, the test group with C/N = 5 was the first to reach 100% ammonia–nitrogen removal. The too low C/N (C/N = 1) or too high C/N (C/N = 20) was not favorable for the growth and the denitrification of the strains. Remaining ammonia–nitrogen at low C/N may be due to the depletion of organic carbon and the inability of S.P-1 to carry out its normal life activities, which results in the ammonia–nitrogen being unable to be further degraded. The low removal rate under excessive C/N conditions was analyzed for the inhibition of related enzymes activity [25] or the accumulation of intermediate products [26]. Combining these results, it was determined that C/N = 5, and we continued with the next experiment.

#### 3.1.3. Effect of Initial pH

**Figure 4:**
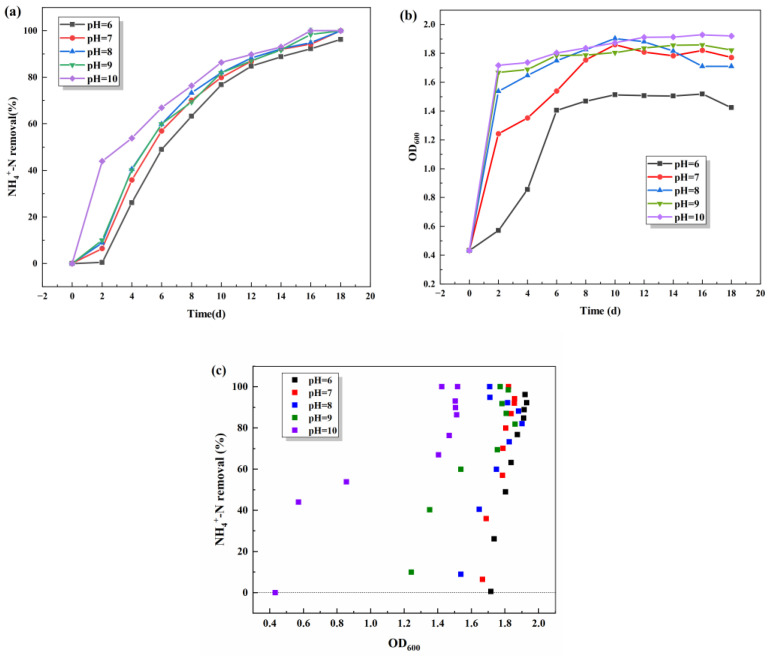
Effects of initial pH on NH_4_^+^-N removal (**a**) and OD_600_ (**b**) and scatter plot of NH_4_^+^-N removal and OD_600_ (**c**).

The effect of the initial pH on the denitrification performance of S.P-1 was investigated by varying the initial pH (pH = 6, 7, 8, 9, 10). From Figure 4, it can be seen that S.P-1 has a good ability to reproduce and remove ammonia–nitrogen under weak alkaline environments (pH = 8, 9, 10). The growth rate and quantity of S.P-1 increase with the increase in pH value, leading to an excellent ammonia–nitrogen removal rate (Figure 4c). The test group with pH = 7~10 has the same concentration of bacteria, the same fastest growth rate, and the same ammonia–nitrogen removal rate. In regard to the number of S.P-1 stabilized in the later stage, the OD600 of the test groups (pH = 7~10) stabilized between 1.6 and 2.0, and the pH = 6 test group, where the OD600 value was around 1.4, indicated that the bacteria are in a good state of growth (pH = 7~10). And 100% ammonia–nitrogen removal was achieved on the 16th day with pH = 7~10. However, ammonia–nitrogen removal was 96.2% on the 16th day with pH = 6. In conclusion, at the initial pH = 8, SP-1 already has a good degradation effect on ammonia wastewater. It indicates that SP-1 can adapt to weakly alkaline environments with pH = 7–10.

Low pH may lead to DNA and protein damage [27]. Most HNAD bacteria prefer a slightly alkaline environment. It is generally believed that more NH_3_ (a direct source of nitrogen for bacterial growth) can be formed under alkaline conditions and thus passively diffuse across the cell membrane. The influx of NH_3_ reduces the metabolic burden and favors the growth of HNAD bacteria compared to NH_4_^+^, which is dependent on energy transport [28,29]. The preference of HNAD bacteria for alkaline environments is not only related to the conversion of NH_4_^+^ to NH_3_, but also to other physiological functions of the bacteria. It has been shown that *Pseudomonas* promotes secondary metabolism (e.g., pigment synthesis) at pH = 7–9 [30]. The suitable environmental pH for most *Pseudomonas* spp. is slightly alkaline, and our results are in agreement with the findings of *Pseudomonas* spp. in alkaline wastewater.

#### 3.1.4. Effect of Inoculum Amounts

**Figure 5:**
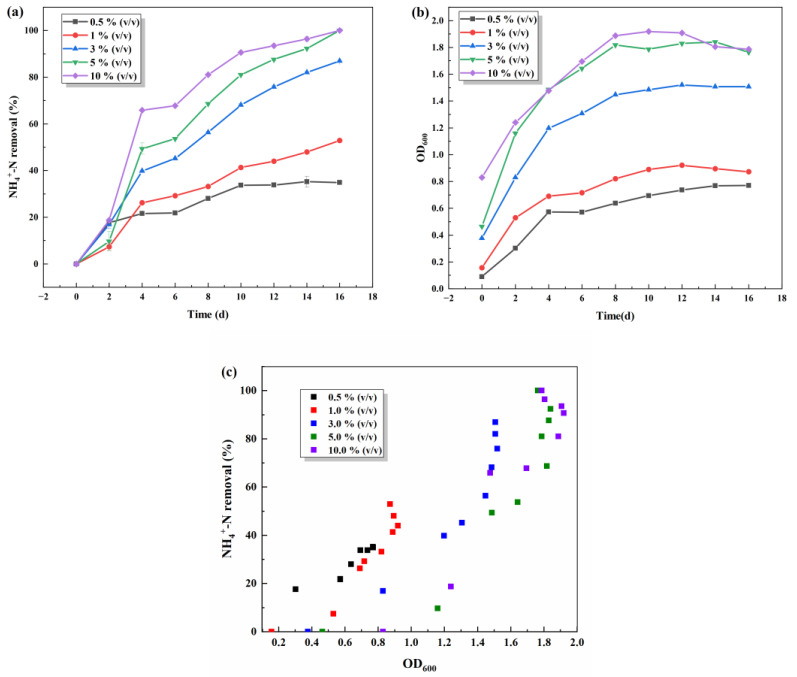
Effects of inoculum amounts on NH_4_^+^-N removal (**a**) and OD_600_ (**b**); scatter plot of NH_4_^+^-N removal and OD_600_ (**c**).

The effect of the inoculum amount on denitrification performance of S.P-1 was investigated by varying the amount of inoculum (0.5%, 1%, 3%, 5%, and 10%) in the wastewater. As can be seen from Figure 5, the density of S.P-1 generally tends to increase and then remain stable as the inoculum volume increases. When the inoculum amount is 5% and 10%, the growth of the strain is better, and the bacterial denitrification capacity is basically maintained at the same level. When the inoculum amount was 0.5%, 1% and 3% made only a low-range bacterial density in the late stage due to the low initial concentration. The highest denitrification rate was achieved within 2–4 day, with ammonia–nitrogen removal increasing from 9.6% to 49.4% in the 5% inoculation group and ammonia–nitrogen removal increasing from 18.7% to 65.9% in the 10% inoculation group. The experimental group inoculated with 0.5% had the lowest bacterial density and the lowest ammonia–nitrogen removal rate. From the graph, it can be judged that there is a positive correlation link between ammonia–nitrogen removal rate and the number of bacteria: the higher the number of bacteria, the greater the ammonia–nitrogen removal rate. After comprehensive analysis, the inoculum quantity of 5% was chosen to continue the subsequent experiment.

### 3.2. Heterotrophic Nitrification–Aerobic Denitrification Capacity of S.P-1

#### 3.2.1. Differences in Ammonia–Nitrogen Removal Efficiency of S.P and S.P-1

**Figure 6:**
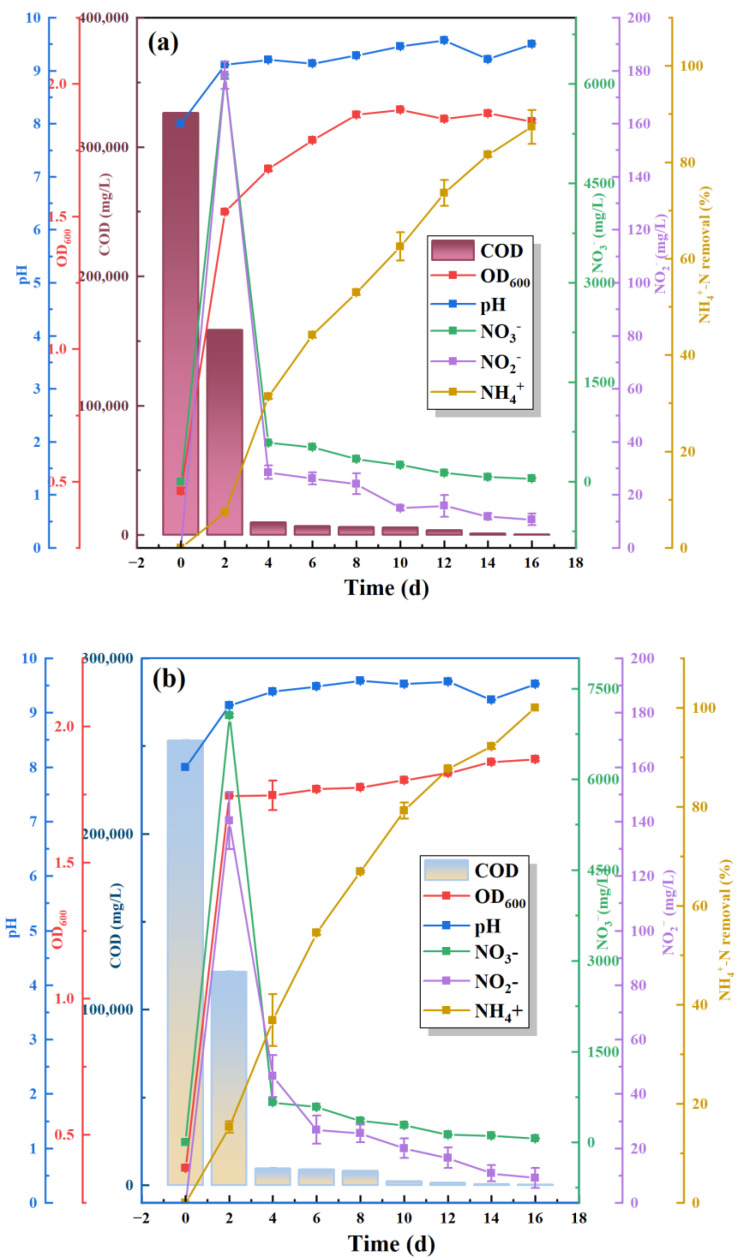
Comparison of heterotrophic nitrification and aerobic denitrification by S.P (**a**) and S.P-1 (**b**).

Sodium citrate is the only carbon source, C/N = 5, pH = 8, and 5% inoculum to explore the differences in growth and removal of ammonia–nitrogen between S.P and S.P-1. As can be seen from Figure 6, the COD concentration in the wastewater treated by the two bacteria decreased rapidly with the extension of time. The pH rapidly increased to between 9 and 9.5 on the second day and then stabilized. The OD_600_ of S.P rapidly increased to 1.5 on the 2nd day and then entered into a stabilization phase. However, S.P-1 is more adapted to the given environment with a rapid increase in OD_600_ to 1.75 on the 2nd day and a stable bacterial population. S.P-1 completely removed ammonia–nitrogen in 16 days, while S.P has an ammonia–nitrogen removal rate of 87.33% at 16 days, so S.P-1 has a 12.7% enhancement in the NH_4_^+^ removal rate compared to S.P. Compared with conventional nitrifying bacteria, S.P-1 strain showed obvious advantages of heterotrophic nitrification performance and high ammonia–nitrogen removal rate. The NO_3_^−^ concentration increased the highest concentration on the 2nd day and then gradually decreased to less than 50 mg L^−1^. The trend of NO_2_^−^ and NO_3_^−^ concentration is the same, and the NO_2_^−^ concentration is less than 10 mg L^−1^ on the 16th day. In general, toxic effects on microorganisms occur at nitrite concentrations greater than 30 mg L^−1^ [31]. He et al. [32] showed that the initial removal of 200 mg L^−1^ nitrite by *Pseudomonas* Y-12 is only 22.4%. In addition, some HNAD-capable bacteria do not have a separate aerobic denitrification function for nitrite removal, such as WY-01 [33]. S.P-1 is able to remove ammonia–nitrogen, nitrate nitrogen, and nitrite nitrogen, and it showed good HN-AD function. Ren et al. [34] showed that when the concentration of ammonia–nitrogen in wastewater exceeded 100 mg L^−1^, the ammonia–nitrogen removal by conventional nitrifying bacteria was significantly reduced, and nitrate and nitrite were accumulated. This may be the result of gene manipulation leading to enhanced ammonia–nitrogen removal by S.P-1.

#### 3.2.2. Evaluation of Nitrification–Denitrification Capacity of S.P-1 at Different Nitrogen Sources

**Figure 7:**
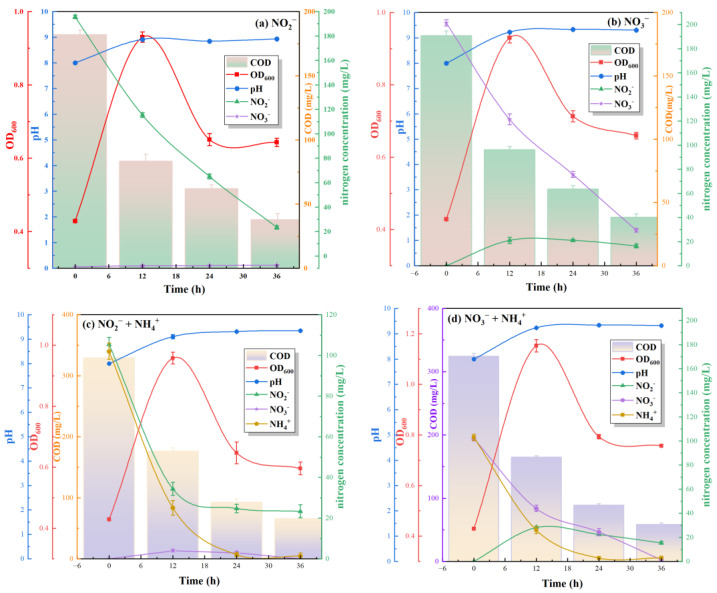
Nitrification–denitrification of S.P-1 under different nitrogen sources.

In order to investigate the denitrification performance of S.P-1 under different nitrogen sources, it is cultured under different nitrogen sources, and the changes of ion content in the solution are recorded (Figure 7). In the single nitrogen source test, when NO_2_^−^ is used as the sole nitrogen source, NO_2_^−^ is reduced from an initial 195.6 mg L^−1^ to 115.2 mg L^−1^ after 12 h, and the degradation rate of NO_2_^−^ is 6.7 mg L^−1^ h^−1^. At the same time, COD decreased from 182 mg L^−1^ to 83 mg L^−1^. It is shown that the strain has the function of simultaneous denitrification and decarbonization. The concentration of S.P-1 increased substantially within 12 h and then decreased slightly and remained stable after 12 h. The degradation process of nitrogen and COD is relatively consistent with the growth of the strains, and the removal rate is higher during the rapid proliferation period. The results are similar for nitrogen source substitution. When NO_3_^−^ is the sole nitrogen source, NO_3_^−^ was reduced from 201.3 mg L^−1^ to 121.3 mg L^−1^ after 12 h, and the degradation rate of NO_3_^−^ was 6.66 mg L^−1^ h^−1^. The denitrification efficiency of S.P-1 is higher than that of many strains used for the treatment of nitrogen-polluted wastewater, such as strain ZJB20129 (1.77 mg L^−1^ h^−1^) [35], strain ND7 (2.77 mg L^−1^ h^−1^) [36], and strain CY-10 (3.03 mg L^−1^ h^−1^) [37]. When S.P-1 treated the mixed nitrogen source, the removal rates of NH_4_^+^ and NO_2_^−^ mixtures were 6.42 mg L^−1^ h^−1^ and 5.91 mg L^−1^ h^−1^, respectively. Similarly, the addition of ammonia–nitrogen changed the removal of NO_3_^−^, with NO_3_^−^ being removed at a rate of 4.83 mg L^−1^ h^−1^ by 12 h. The removal rate of S.P-1 was slightly delayed at the mixed nitrogen source than at the single nitrogen source, probably due to the production of new nitrate and nitrite nitrogen during the degradation of NH_4_^+^.

In the nitrification pathway with NH_4_^+^ as the sole nitrogen source, NH_4_^+^ undergoes sequential oxidation via ammonia monooxygenase (AMO) to hydroxylamine (NH_2_OH), followed by further conversion to nitrite (NO_2_^−^) and nitrate (NO_3_^−^). As AMO is an obligately aerobic enzyme requiring molecular oxygen for catalytic activity, all cultures were maintained under orbital shaking (200 rpm) to ensure sufficient oxygen supply, consistent with aerobic operational parameters. When NO_3_^−^/NO_2_^−^ is the only source of nitrogen (denitrification), NO_3_^−^ is reduced to NO_2_^−^ by nitrate reductase (NarG/NapA), and NO_2_^−^ is further converted to NO by nitrite reductase (NirS). Although conventional denitrification requires anoxic conditions, the S.P-1 in this study belonged to the HNAD strain, which showed aerobic denitrification ability, and the activities of NarG and NirS remained functional (successful amplification of narG and nirS genes in Table 4). With a mixed nitrogen source (NH_4_^+^+NO_3_^−^/NO_2_^−^), the oxidation of NH_4_^+^ under aerobic conditions (nitrification) was synchronized with the NO_3_^−^/NO_2_^−^ reduction (denitrification) in parallel (Figure 7). Oxygen regulates this process through a dual action; first, it enhances AMO enzyme activity (Figure 14) and accelerates NH_4_^+^ oxidation. Second, S.P-1 successfully encodes the napA gene (Table 4) to overcome the competitive inhibition of the denitrifying enzyme activity caused by oxygen.

### 3.3. Mechanism of Simultaneous Nitrification–Denitrification Removal of Ammonia–Nitrogen by S.P-1

#### 3.3.1. Identification of Strain S.P-1

**Figure 8:**
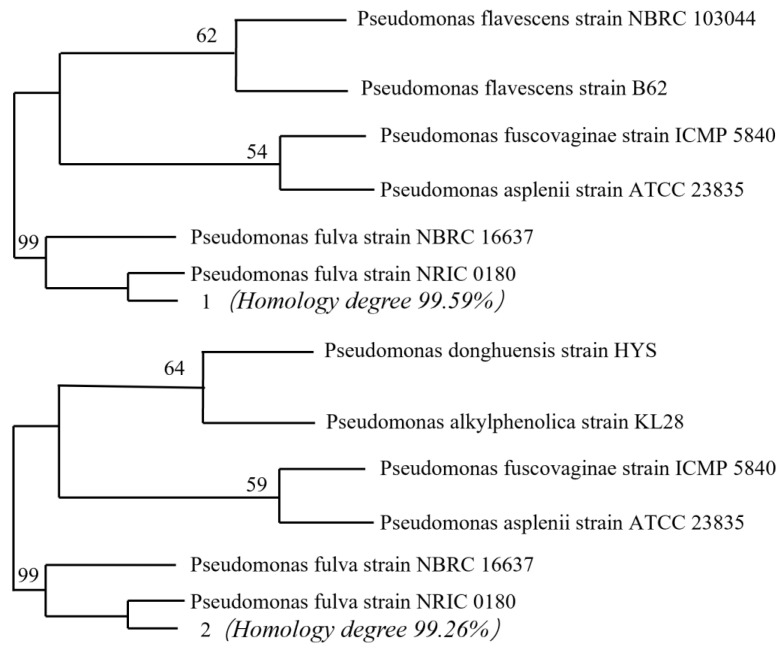
Developmental tree of S.P and S.P-1.

In Figure 8, sequencing of the 16S rRNA gene and BLAST analysis showed that the strain purchased from CGMCC is 99.59% homologous to the *Pseudomonas* fulva strain NRIC 0180, and the domesticated strain showed 99.26% homology with *Pseudomonas* fulva strain NRIC 0180. Both strains are most closely related to the genus *Pseudomonas* and can be categorized as *Pseudomonas* sp. It suggests that the genus of bacteria do not change and do not mutate into other species after domestication in vanadium-containing solutions.

#### 3.3.2. Changes in Extracellular Polymeric Substances (EPS) of S.P-1

**Figure 9:**
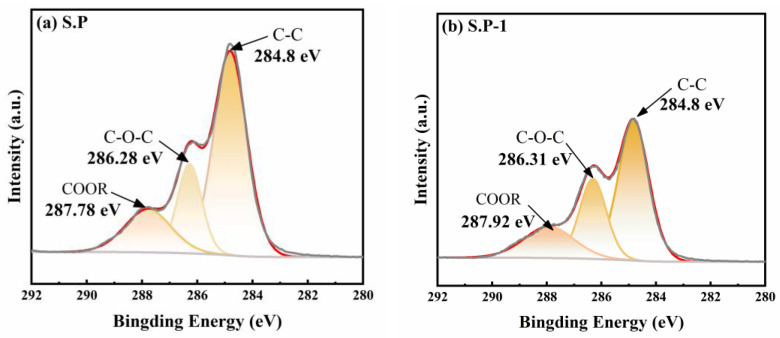

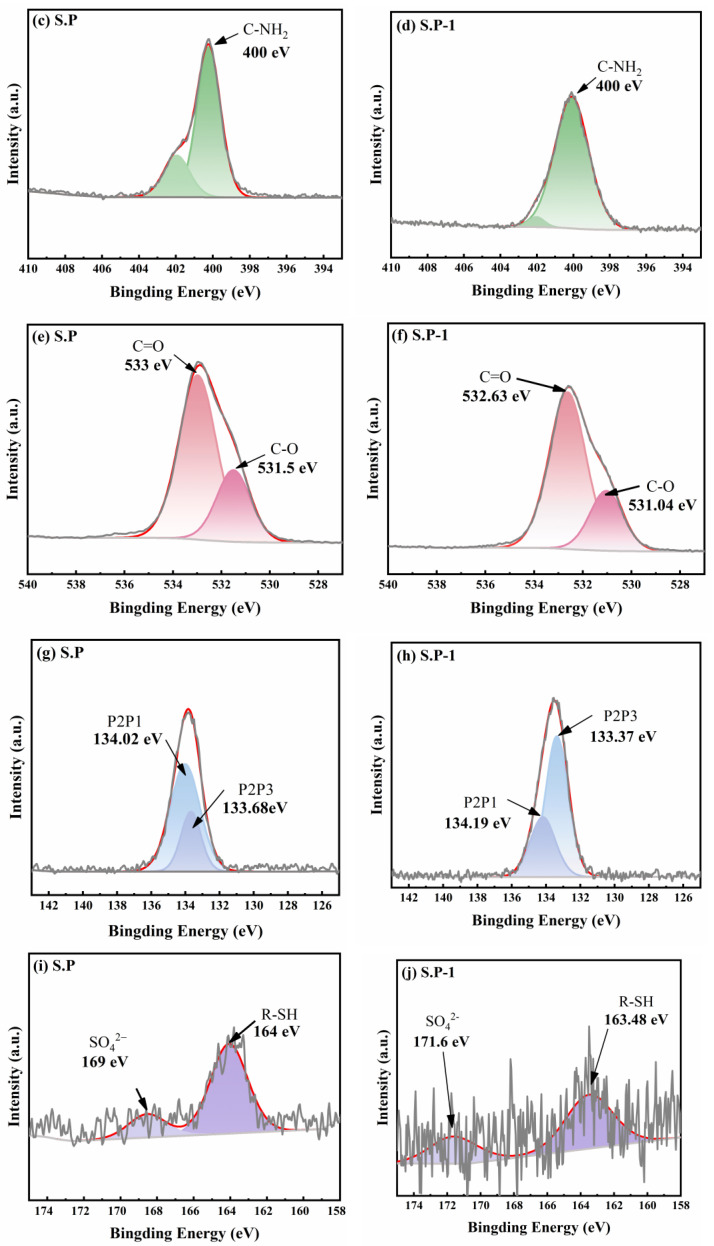
The C (**a**,**b**), N (**c**,**d**), O (**e**,**f**), P (**g**,**h**), and S (**i**,**j**) XPS energy spectrum of S.P and S.P-1.

To analyze the effect of vanadium stress on the extracellular polymer (EPS) fractions of S.P-1, the EPS powders were subjected to full-spectrum and high-resolution scans of C 1s, N 1s, O 1s, P 2p, and S 2p using a ThermoScientific K-Alpha XPS instrument (ThermoScientific, Waltham, MA, USA), and the peak areas were deconvolved and analyzed semi-quantitatively by Avantage software 6.0. As shown in the Figure 9, the C1s peaks of both S.P and S.P-1 are decomposed into three different peaks, each with a different peak area, indicating that their contents are different. The three peaks of the S.P are C-(C,H) (284.8 eV), C-(O, N) (286.28 eV), and C = O or O-C-O (287.78 eV). The three peaks for the S.P-1 are C-(C, H) (284.8 eV), C-(O, N) (286.31 eV), and C=O or O-C-O (287.92 eV). C-(C, H) comes mainly from a large portion of the side chains of polysaccharide lipids or amino acids. C-(O, N) comes mainly from alcohols, amines, or ether amides and C=O or O-C-O from carboxylates, carbonyls, and amides [38]. After comparing the changes in functional group content before and after domestication, C-(C, H) increased from 2.77% to 33.59%, C-(O, N) increased from 1.83% to 17.25%, and C=O or O-C-O increased from 1.68% to 19.62% after domestication. Carbon–nitrogen bonds and amide groups of the class are often found in proteins [39], and O-C-O is an important functional group in polysaccharides. These results suggest that domestication plays an important role in the changes of polysaccharides and proteins on the surface of bacteria. It has been reported that exogenous stress stimulates the strain to secrete more proteins and other substances for its own protection and nutritional replenishment [40].

Two main states of elemental N exist in S.P and S.P-1: the amino group (-NH_2_) located near 400 eV and the protonated amino group (-NH_3_^+^) located near 402 eV. As can be seen from the Figure 8, the relative contents of both amino (-NH_2_) and protonated amino (-NH_3_^+^) changed before and after domestication, with the relative content of amino (-NH_2_) increasing from 0.7% to 8.41% and the relative content of protonated amino (-NH_3_^+^) increasing from 0.21% to 0.3%. The S.P formed C=O bonds at 533 eV and C-O bonds at 531.5 eV, and the domesticated bacteria formed C=O bonds at 532.63 eV and C-O bonds at 531.5 eV; before and after domestication, the relative C=O content increased from 2.26% to 12.58%, and the relative C-O content increased from 0.9% to 6.75%. The relative content of elemental P increased from 0.14% to 1.4%, and the relative content of elemental S increased from 0.02% to 0.09% after domestication.

The main components in EPS are polysaccharides, lipids, and proteins. The relative content of polysaccharides (PEP) and proteins (PS) on the cell surface can be estimated by the following equation [41]:

(1) $$\frac{N}{C}=0.279(\frac{Cpn}{C})$$

(2) $$\frac{O}{C}=0.325\left(\frac{Cpn}{C}\right)+0.833(\frac{Cps}{C})$$

(3) $$\frac{C}{C}=\left(\frac{Cpn}{C}\right)+\left(\frac{Cps}{C}\right)+(\frac{Chs}{C})$$

O/C and N/C are the observed concentration ratios of oxygen and nitrogen atoms, respectively, relative to the concentration ratio of carbon atoms in the analyzed sample. Cps, Cpn, and Chc are the atomic concentrations of carbon present in polysaccharides, proteins, and hydrocarbon-like products, respectively. Based on the above equations, the proportions of the major carbon-related components in the three EPS samples are reported as shown in Table 3.

EPS provide a moist environment for microorganisms to capture nutrients, facilitate chemical reactions, and protect cells from stressful environmental conditions. After calculation, it was found that the polysaccharide content increased by 11.34%, and the protein content increased by 37.94% of S.P-1 compared to S.P. In addition, the positions and shapes of the characteristic peaks in the X-ray photoelectron spectra basically do not change significantly, but the intensities of the characteristic peaks changed, indicating that the chemical structure of EPS did not change, while the relative contents of polysaccharides and proteins composed of these elements changed. Both proteins and polysaccharides are key functional components of EPS [42,43]. The proteins of EPS contain a large number of negatively charged functional groups, such as C=O, N-H, -COO-, C-N, -OH, etc., [44,45], indicating an increase in the relative content of negatively charged functional groups of S.P-1. EPS can help to adsorb and bind more NH_4_^+^ in an aqueous solution by electrostatic interactions [40].

#### 3.3.3. Effects of Ammonia and Vanadium on the Membrane Potential and Permeability of *Pseudomonas* Aeruginosa Cells

**Figure 10:**
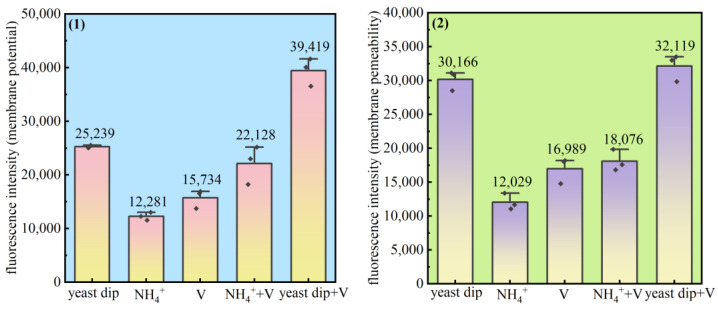
The potential difference between inside and outside the membrane (**1**) and membrane permeability (**2**) of *Pseudomonas* aeruginosa under different ammonia and vanadium conditions.

*Pseudomonas* sp. was cultured in an organic nitrogen source (yeast dip), an inorganic nitrogen source (NH_4_^+^), an organic nitrogen source and vanadium (yeast dip and V), an inorganic nitrogen source and vanadium (NH_4_^+^+V), a vanadium-only basal medium were used for domestication and passaging. The membrane potential and membrane permeability of the bacteria under each condition were analyzed, and it can be seen from Figure 10 that the potential difference between the inside and outside of the membrane and the permeability of each bacterium showed a similar trend; the combination of yeast dip and V had the highest potential difference between the inside and outside of the membrane and the highest permeability, and the fluorescence intensity values of the inside and outside of the membrane of the various groups were arranged as organic nitrogen source and V (yeast dip and V) > organic nitrogen source (yeast dip) > inorganic nitrogen source and V (NH_4_^+^ and V) > V > inorganic nitrogen source (NH_4_^+^). The decrease or loss of membrane potential predicted that the bacteria were affected by the action of external substances, and there was a close relationship between the cell membrane potential and membrane permeability, and the change of membrane potential could affect the permeability of the membrane [46,47], which in turn could affect the transmembrane transport of substances and other physiological functions. It affected the ability of *Pseudomonas* sp. to transport ammonia–nitrogen. With the intervention of vanadium, the bacterial membrane potential difference and permeability were increased, which contributed to the release of metabolites and nutrient translocation within the bacterium, and the bacterial material transport capacity was enhanced.

#### 3.3.4. S.P-1 Hydrophilic Enhancement

**Figure 11:**
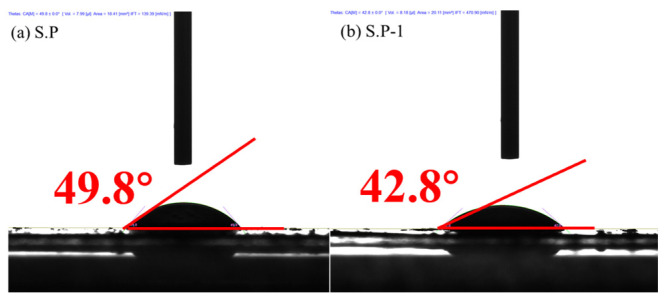
Contact angle of S.P (**a**) and S.P-1 (**b**).

The contact angle of the bacterial surface is an important parameter describing the wetting properties of the bacterial surface and is closely related to the hydrophobicity of the bacterial surface. It can be seen from Figure 11 that the contact angle of S.P-1 is less than that of S.P. This indicates that S.P-1 has better wettability and good hydrophilicity. This may be due to the increase in negatively charged functional groups in EPS, which has an enhanced ability to attract positively charged hydrogen atoms in water molecules and interact with water more easily [48].

#### 3.3.5. Accumulation Behavior of Vanadium in S.P-1

**Figure 12:**
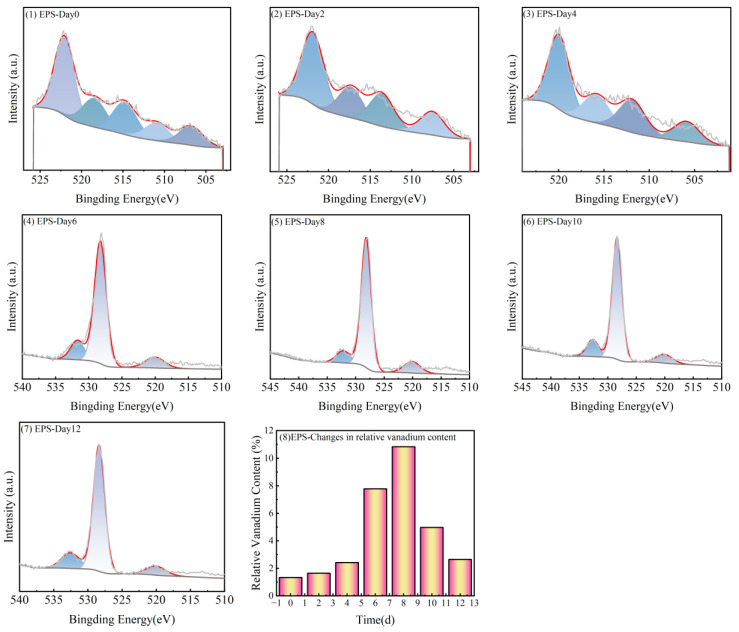
Passive adsorption of vanadium by S.P-1.

The biological interactions between microorganisms and heavy metal ions mainly include complex mechanisms such as microbial-mediated adsorption, leaching, transfer, degradation, mineralization, and bioaccumulation, which are broadly classified as passive and active adsorption [49]. “Passive adsorption” refers to the non-metabolic physicochemical binding of substances by microorganisms through extracellular polymers (EPS) or functional groups on the cell surface. This process does not require cellular energy expenditure, and surface complexation occurs primarily through electrostatic interactions or ligand exchange [50]. A large number of EPS existed outside the biofilm of microbial cells, containing a variety of reactive groups, which could adsorb heavy metal ions on the surface of the biofilm through passive processes. The changes of the relative content of vanadium in S.P-1 EPS are shown in Figure 12. The relative content of vanadium in EPS showed a trend of increasing and then decreasing, with the highest relative content of 10.82% at day 8, when the S.P-1 growth reached the stabilization period.

**Figure 13:**
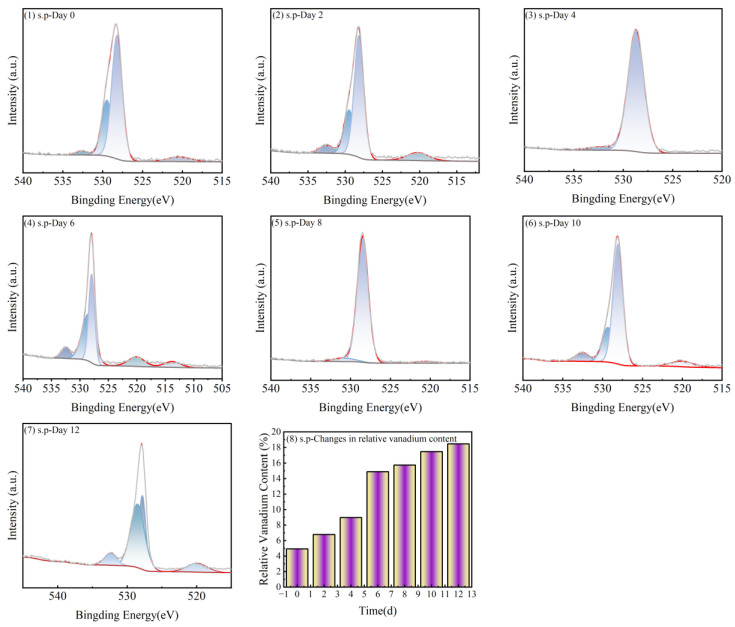
Active adsorption of vanadium by S.P-1.

Heavy metal biosorption by microorganisms consists of two steps; in addition to the passive adsorption of heavy metal ions, which is not related to metabolism, there is also an active biosorption (bioaccumulation), which mainly refers to the uptake of metals (transport to the cell, intracellular accumulation, and transcellular membranes through cellular metabolic cycling) by living cells through a slower active metabolism, which relies on the translocation of heavy metals into bacterial cells [51]. When the cell membrane adsorption of heavy metals reaches a certain amount of adsorption, the heavy metals will enter the microbial cell to undergo the accumulation process [52]. In Figure 13, the relative content of vanadium in S.P-1 showed a trend of first increasing and then gradually stabilizing, and the relative content of vanadium in S.P-1 was 18.45% on the twelfth day, and the vanadium adsorbed passively by the EPS was gradually transferred to the interior of the bacterial organism, and the vanadium accumulation phenomenon occurred in the organisms.

#### 3.3.6. Enhancement of Internal Nitrification Process in S.P-1

**Figure 14:**
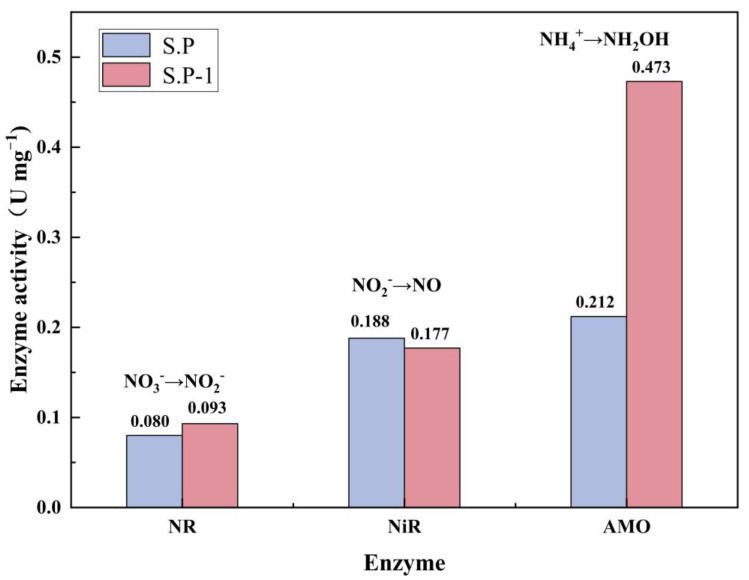
Specific enzyme activities of NR, NiR, and AMO enzymes in S.P and S.P-1.

Generally speaking, the heterotrophic-nitrifying bacteria, producing N_2_ via an aerobic deammonium pathway, is mainly divided into two kinds: One is the ammonium as the substrate of a heterotrophic nitrification reaction coupled with nitrate (or nitrite) as the substrate of aerobic denitrification reaction. The other is the direct conversion of NH_4_^+^ to N_2_ through NH_2_OH. To understand the possible denitrification pathways of S.P-1, the activities of four key enzymes, NiR, NR, AMO and HAO, are studied under aerobic conditions (Figure 14). NR and NiR are two key enzymes associated with denitrification. NR is a nitrate reductase that catalyzes the reduction in nitrate to nitrite by NAD(P)H. NiR is a synthetic nitrite reductase encoded by the nirS and nirK genes that catalyzes the reduction in nitrite. The NR- and NiR-specific enzyme activities are 0.08 U mg^−1^ and 0.188 U mg^−1^ for S.P and 0.093 U mg^−1^ and 0.177 U mg^−1^ for S.P-1, respectively. The specific enzyme activities of both S.P and S.P-1 are in the same order of magnitude. The NR-specific enzyme activity of S.P-1 increased by 16.25%, and the NiR-specific enzyme activity decreased by 5.85% as compared to S.P. The presence of NR and NiR explains the near-absence of nitrate and nitrite accumulation during deammoniation, providing preliminary evidence that the S.P-1 heterotrophic nitrification reaction couples an aerobic denitrification reaction with nitrate (or nitrite) as a substrate. AMO is an ammonia monooxygenase that catalyzes the conversion of ammonia–nitrogen to hydroxylamine and is often considered the rate-limiting enzyme in heterotrophic nitrification [53]. The specific enzyme activities of S.P and S.P-1 are 0.212 U mg^−1^ and 0.473 U mg^−1^, respectively. The specific enzyme activity of AMO in S.P-1 is significantly higher than that of other enzymes involved in nitrogen metabolism, providing a direct conversion of ammonium to N_2_ through hydroxylamine preliminary evidence. HAO is a hydroxylamine oxidoreductase that catalyzes hydroxylamine to nitrate in a specific manner, but it was not detected, as observed in the other published literature [54,55]. It may be that the level of this enzyme is too low to be easily detected, or it may be that there is an unknown enzyme at work. A study [56] has purified pyruvate oxime dioxygenase (POD) from Alkaliella faecalis oxygenase (POD) and catalyzed the oxidation of NH_2_OH to NO_3_^−^-N.

To further illustrate the metabolic pathways of ammonia and nitrogen in the strains, the PCR amplification of genes related to nitrogen metabolism (napA, napG, nirS, norB, amoA, and nosZ) in S.P and S.P-1 was performed, and the results are shown in Table 4. Based on the DNA level, amplification of the amoA gene in S.P yielded an approximately 145 bp product, and amplification of the amoA gene in S.P-1 yielded an approximately 362 bp product. The amoA is a gene encoding an ammonia monooxygenase that catalyzes the specific conversion of NH_4_^+^ to NH_2_OH. The amoA gene amplification product is much higher in S.P-1 than in S.P. The gene sequence is changed as a result of bacteria adapting to a new vanadium-containing environment during domestication. It is also consistent with the significantly higher AMO-specific enzyme activity in S.P-1, where an increase in the gene sequence led to an increase in the specific enzyme activity encoding the corresponding enzyme. The amplified product of the napA gene is 129 bp and is involved in the conversion of NO_3_^−^ to NO_2_^−^. It is insensitive to oxygen molecules and is often regarded as a signature gene for nitrate respiration in the presence of oxygen. It is often used as a marker to identify HNAD strains. The amplification product of the narG gene is approximately 148bp and usually plays a major role in anaerobic denitrification. Successful amplification of both genes shows that both forms of the nitrate reductase genes can coexist in a single strain [57] and encode the NR catalyzing the conversion of NO_3_^−^ to NO_2_^−^ [35], suggesting the denitrification ability of S.P-1 [58]. The nirS plays an important role in denitrification and is a key gene encoding nitrite reductase (NiR), which catalyzes the conversion of NO_2_^−^ to NO [59], and a 131 bp amplification product is obtained in S.P-1. In addition, amplification products of 142 bp and 123 bp are successfully obtained for norB and nosZ genes, indicating that S.P-1 follows the conversion pathway of NO_2_^−^-N→NO→N_2_O→N_2_ during denitrification. The denitrification curve demonstrates that the rate of ammonia–nitrogen removal by S.P-1 is superior to that of nitrate nitrogen and nitrite nitrogen. The specific enzyme activity of AMO relating to the nitrification process is greater than that of NR and NiR, indicating that under the domestication of vanadium-containing solutions, the ability of S.P-1 nitrification is stronger than that of denitrification, and the expression of amoA functional genes is stronger than that of napA functional genes.

Based on the PCR amplification of genes related to nitrogen metabolism, the pathway for removing NH_4_^+^ within S.P-1 is the following: NH_4_^+^ is converted to NH_2_OH by the catalytic action of amo-coded AMO, and NH_2_OH is converted to NO_3_^−^ under specific action. The generated NO_3_^−^ is converted to NO_2_^−^ under the NR co-encoded by napA and napG, and later generates NO. NO is converted to N_2_O under the regulation of norB, and N_2_O is finally converted to N_2_ for excretion under the regulation of nosZ.

#### 3.3.7. Mechanism of S.P-1 Enhanced Ammonia–Nitrogen Removal

**Figure 15:**
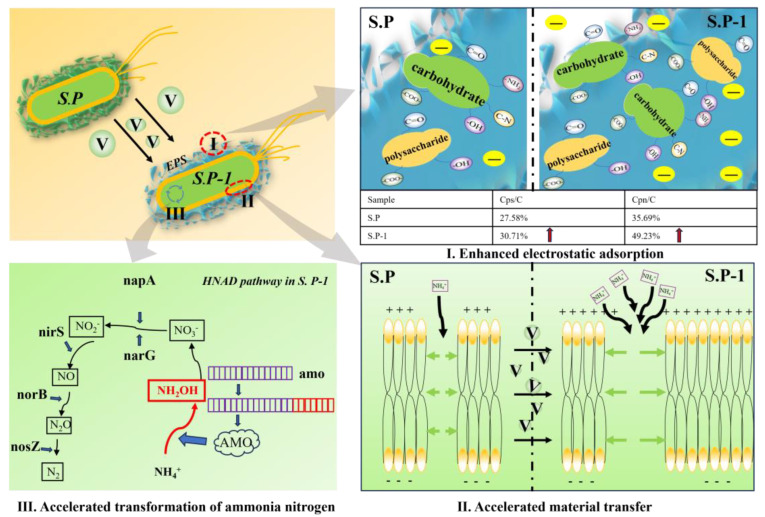
Mechanism of S.P-1-enhanced ammonia–nitrogen removal.

Under the stimulation of vanadium-containing solutions, vanadium enters S.P-1 by passive and active adsorption, stimulating S.P-1 to induce changes in the secretion of EPS through gene regulation (Figure 15). The relative content of polysaccharides and proteins in the EPS of S.P-1 increased, in which the relative content of negatively charged functional groups such as C=O, N-H, -COO-, C-N, -OH, etc., increased, and this resulted in decreases in the S.P-1 contact angle and increases in hydrophilicity. The ability of negatively charged functional groups to adsorb NH_4_^+^ electrostatically is enhanced, and EPS adsorbs many positively charged substances. The potential difference between the inside and outside of the cell membrane increased. In order to maintain the normal life activities of the bacteria, the permeability of the cell membrane to specific ions changed, which contributed to the release of metabolic substances within the bacterium and the uptake of nutrients from the outside world, and facilitated the transportation of NH_4_^+^ into the cell by S.P-1. Meanwhile, domestication in vanadium-containing solutions promotes changes in the sequence of the amo gene, which directs the transition from NH_4_^+^ to NH_2_OH in bacteria, and was altered, with an increase in base pair length and an increase in the activity of the amo-encoded AMO enzyme, indicating up-regulation of amo gene expression and accelerating the process of conversion of NH_4_^+^ to NH_2_OH. PCR successfully amplified napA, napG, nirS, norB, amoA, and nosZ, indicating that these genes are present in S.P-1 and satisfy the HNAD pathway for NH_4_^+^ removal: NH_4_^+^→NH_2_OH→NO_2_^−^→(NO_3_^−^→NO_2_^−^→)NO→N_2_O→N_2_.

## 4. Conclusions

This study utilized vanadium-containing solutions to domesticate *Pseudomonas* sp. and used biological methods to remove high concentration ammonia–nitrogen (pH = 8, 2000 mg L^−1^) from vanadium-containing wastewater in the vanadium-bearing shale vanadium extraction industry. *Pseudomonas* sp. (S.P) is domesticated in vanadium-containing wastewater to obtain domesticated *Pseudomonas* sp. (S.P-1). Sodium citrate was the external carbon source, the C/N ratio was 5, the bacterial inoculation amount was 5%, and initial wastewater pH was 8. S.P-1 removed more than 99.25% of ammonia in wastewater after 16 days, and S.P-1 had a 12.7% enhancement in the NH_4_^+^-N removal rate compared to S.P.

Intervention of vanadium entry into S.P-1 stimulated an increase in polysaccharide and protein content in the extracellular polymeric substance (EPS) by 11.34% and 37.94%, respectively. The relative content of negatively charged functional groups increased, resulting in the enhancement of hydrophilicity and positively charged substances’ (e.g., NH_4_^+^) adsorption capacity. In addition, the increase in potential difference between the inside and outside of the cell membrane and membrane permeability leads to an increase in material transport efficiency.

Vanadium acts as a stimulant in the bacterial enzyme system, stimulating an increase in the activity of specific enzymes of AMO, thereby accelerating the transition from NH_4_^+^ to NH_2_OH. The functional genes (napA, napG, nirS, norB, amoA, and nosZ) are successfully amplified by PCR, which indicates that it has both nitrification and denitrification capabilities. The amoA-amplified products increased in length to enhance nitrification capacity, and the S.P-1 nitrification capacity was stronger than the denitrification capacity. The degradation process of NH_4_^+^ in S.P-1 is the following: NH_4_^+^→NH_2_OH→NO_2_^−^→(NO_3_^−^→NO_2_^−^→)NO→N_2_O→N_2_.

It was revealed that vanadium is not only a toxic pollutant but also a functional driver of microbial adaptation. By combining vanadium stress with targeted physiological changes (EPS synthesis and AMO up-regulation) in bacteria, we simultaneously overcame the dual problems of NH_4_^+^-N accumulation and vanadium toxicity in wastewater recirculation and avoided the secondary pollution (waste gas and waste residue) produced by the traditional materialization method (such as blowing off and chemical precipitation) and provided a strategy for wastewater remediation. Future research could focus on improving the economics of the technology, such as exploring agricultural or food industry waste as a low-cost carbon source, to further reduce the cost of the technology when it is actually in operation.

## Figures and Tables

**Table 1:** Elemental composition of ammonia–nitrogen wastewater.

Elemental	Mg	Na	Ca	K	V	N-NH_4_^+^
concentration (mg L^−1^)	18.45	1533.82	101.36	77.49	96.00	1999.56

**Table 2:** List of gene-specific primers.

Gene Name	Sequence (5′ to 3′)
16S-F	CGCAGAACCTTACCAACCCT
16S-R	GAGTGCCCAACCAAATGCTG
napA-F2	GGGCTTGAAGACGATGGGAA
napA-R2	ATGCATCCGATCCTGTGGAC
narG-F2	TCATTCCGCTGCCTCCATAC
narG-R2	CCAGTTGGCCTATGGCTTCA
nirS-F3	ACAACCTCAAGACCACCGAGA
nirS-R3	GCCTTCCTTGGTGTCGATCA
norB-F2	CTAGTGCTGCTGTGGCTCTT
norB-R2	TGAGCATCAGGAAGGCAAGG
amoA-F2	GAACAGCGGATAACCGACCA
amoA-R2	CATCTTCATGCGCACCATCC
nosZ-F	AGCGGTCCTTCGAGAACTTG
nosZ-R	ACCCGATCAAGGACAAGCTG

**Table 3:** Percentage content of three major components of bacterial EPS before and after domestication of SP.

Samples	Cps/C (%)	Cpn/C (%)	Chc/C (%)
SP.	27.58	35.69	36.73
SP-1	30.71	49.23	20.06

**Table 4:** Length of PCR amplification products of S.P and S.P-1.

Name	16S-F (bp)	amoA (bp)	napA (bp)	napG (bp)	nirS (bp)	norB (bp)	nosZ (bp)
S.P	177	145	129	148	131	142	123
S.P-1	177	362	129	148	131	142	123

## Data Availability

The original contributions presented in this study are included in the article. Further inquiries can be directed to the corresponding authors.
